# A protocol for ongoing systematic scoping reviews of World Trade Center Health research

**DOI:** 10.1186/s13643-023-02318-x

**Published:** 2023-10-10

**Authors:** Thomas W. Concannon, Ramya Chari, Justin Lee, Liisa Hiatt, Laura J. Faherty

**Affiliations:** 1 The RAND Corporation, 20 Park Plaza Suite 920, Boston, MA USA; 2 Tufts University School of Medicine, Boston, MA USA; 3 The RAND Corporation, Arlington, VA USA; 4 The RAND Corporation, Santa Monica, CA USA

**Keywords:** World Trade Center, Disaster, 9/11 attacks, Translational research, Health

## Abstract

**Background:**

The World Trade Center (WTC) Health Program (“Program”) seeks to assess the inventory, quality, and impact of its funded research in the context of all clinical and translational research involving WTC populations. This paper presents a protocol for ongoing scoping reviews of WTC-related health research.

**Methods:**

Using terms relevant to the September 11 attacks, we will search OVID MEDLINE, PsycINFO, Scopus, Web of Science, CINAHL, and Embase for records of peer-reviewed publications. Title, abstract, and full text screening will be used to exclude records according to a priori criteria. Data abstraction will be performed on all articles that meet inclusion criteria using a standardized query form that was developed in collaboration with NIOSH. A team of reviewers will be trained to abstract data from included articles. Articles will be double-reviewed, and disagreements will be adjudicated.

**Results:**

We will summarize existing research involving WTC populations. The summary will assess the extent, nature, and signals of impact of WTC-related health research.

**Conclusions:**

Our review will lay the groundwork for additional study of research impact by identifying population, clinical, and translational topics that can be assessed through future focused reviews. It will also support planning activities by Program policy makers and stakeholders as they work to achieve the Program’s research goals.

**Systematic review registration:**

This publication serves as documentation of the protocol.

**Supplementary Information:**

The online version contains supplementary material available at 10.1186/s13643-023-02318-x.

## Background

In 2017, the World Trade Center Health Program (hereafter, “Program”) sought to better understand the state of health research involving 9/11 responders and survivors. To respond to this need, we published a comprehensive scoping review of clinical and translational research on 9/11 populations [1]. The scoping review involved the collection of 160 data points on each of nearly 1000 peer reviewed research articles published from September 11, 2001, through October 30, 2020. We found that WTC-related health research addressed a range of health conditions and was balanced in terms of survivor and responder populations. It was concentrated on establishing links between 9/11 exposures and health conditions while concentrating less so on developing health interventions and services for treatment of WTC-related conditions.

Evidence from that review has since been used in scientific planning by the Program in several ways. The review was used to support new funding for interventional and health services research to improve health care delivery and outcomes; further inquiry into special topics such as a focused review and scientific planning meeting on youth-focused research^1^; and direct public access to peer-reviewed research findings [2]. For instance, a publications dashboard scheduled for release in 2023 [3] will offer the public more extensive information on the extent and nature of WTC-related populations, health conditions, clinical interventions, health care quality, and outcomes.

In this article, we present a protocol for future, ongoing scoping review updates. As the Program is authorized to fund medical monitoring, care, and research through 2090, continued growth in published research on WTC-related health can be anticipated, and ongoing scoping review updates will be needed. Ongoing reviews will support scientific research policy, planning, and evaluation over time. They will also support direct public access to research evidence and lay the groundwork for additional focused study of WTC-related research impacts.

Research questions guiding ongoing scoping review updates include the following: (1) What has been studied with respect to the health effects of 9/11 since the initial review was completed?^2^ (2) What signals of research impact can be documented through “mapping” WTC health-related research to recognized frameworks for translational research? [4–6] (3) How is WTC health research impact evolving over time?

## Methods

The updated, ongoing review will encompass evidence from any English language peer-reviewed report of research conducted on or about the health of WTC populations, including case reports, observational studies, controlled trials, and systematic or narrative reviews. The research may be funded by the Program or by other sponsors and may be published between November 1, 2020, and December 31, 2022. Our approach to completing the record search, eligibility screening, data abstraction, analysis, and evidence map of WTC clinical and translational research is described below. In our previous work [1], we validated the search and screening approach by comparing results to lists of pertinent studies identified by topic experts and the “Summary of World Trade Center Health Program Research: NIOSH Research Compendium” [7] completed in 2019 by program staff.

### Record search

We will use a systematic and validated search strategy to find records of WTC-related health research from databases of peer reviewed publications. An information specialist (Larkin) was involved in developing and validating the strategy. We will group search terms into four hierarchical statements, shown below. A full list of search terms is presented in Additional file 1.

1. Terms related to the terrorist attacks on September 11, 2001
2. Terms related to the numerical date (i.e., 9/11) and the type of disaster
3. Terms related to the New York City and New Jersey locations and the type of disaster
4. Terms related to the Shanksville, Pennsylvania, and Pentagon locations and the type of disaster

To find records of peer-reviewed publications, we will search six databases using terms relevant to the September 11 attacks. Using the four statements described above and in Additional file 1, we will search titles, abstracts, and keywords in OVID MEDLINE, PsycINFO, Scopus, Web of Science, CINAHL, and Embase for records of peer-reviewed articles, books, book chapters, conference abstracts, and dissertations.

Validation results, such as recall rates, will be reported in the manuscript. Automated exclusions for non-health research (such as construction engineering studies) will be applied. Included records will be limited to articles published over a 2-year period, from November 1, 2020, through December 31, 2022.

### Eligibility screening

We (Concannon, Faherty, Chari) will apply five exclusion filters of increasing specificity. The filters are as follows: (1) not in English; (2) not peer reviewed research; (3) not about 9/11 attacks; (4) not about 9/11 populations; (5) not about health conditions, care, or outcomes. Titles and abstracts collected during the record search will be screened first. Records that pass sequentially through all five screens will be included in the data abstraction process described below. Those that do not will be excluded and reported as such in a Preferred Items for Reporting on Systematic Reviews and Meta Analyses (PRISMA) flowchart [8]. The first reason for exclusion will be recorded in Supplementary material. Records that are deemed uncertain will proceed to full text screen. In this stage, full text articles will be obtained, and the screening process will be repeated until every record is determined to be eligible or ineligible for inclusion. Final results of the screening process will be reported in the PRISMA flowchart.

### Data abstraction

A standardized query form, shown in Additional file 2, was developed in collaboration with NIOSH to guide data abstraction. Included query term items will be coded into DistillerSR [9] as a survey tool with drop down, numeric text, and free text fields response options. Experienced reviewers (Concannon, Faherty, Chari) will abstract data from included articles. The first 10% of included articles will be double reviewed and adjudicated by the whole team. Single reviews will commence on subsequent rounds of 10% of included articles if 85% agreement is achieved on priority items in the survey tool on at least one of the rounds.

### Inventory of research (RQ1)

An inventory of research (RQ1) will be captured by categorizing research publications according to elements of the PICOTSS and PECOTSS frameworks (population, interventions or exposures, comparators, outcomes, timing, setting, and study design). PECOTSS is the environmental equivalent of the Agency for Healthcare Research and Quality’s PICOTS expansion of the PICO framework, which was developed for evaluations that focus on clinical interventions rather than exposures [10].

### Evidence of research impacts (RQs 2 and 3)

**Fig. 1:**
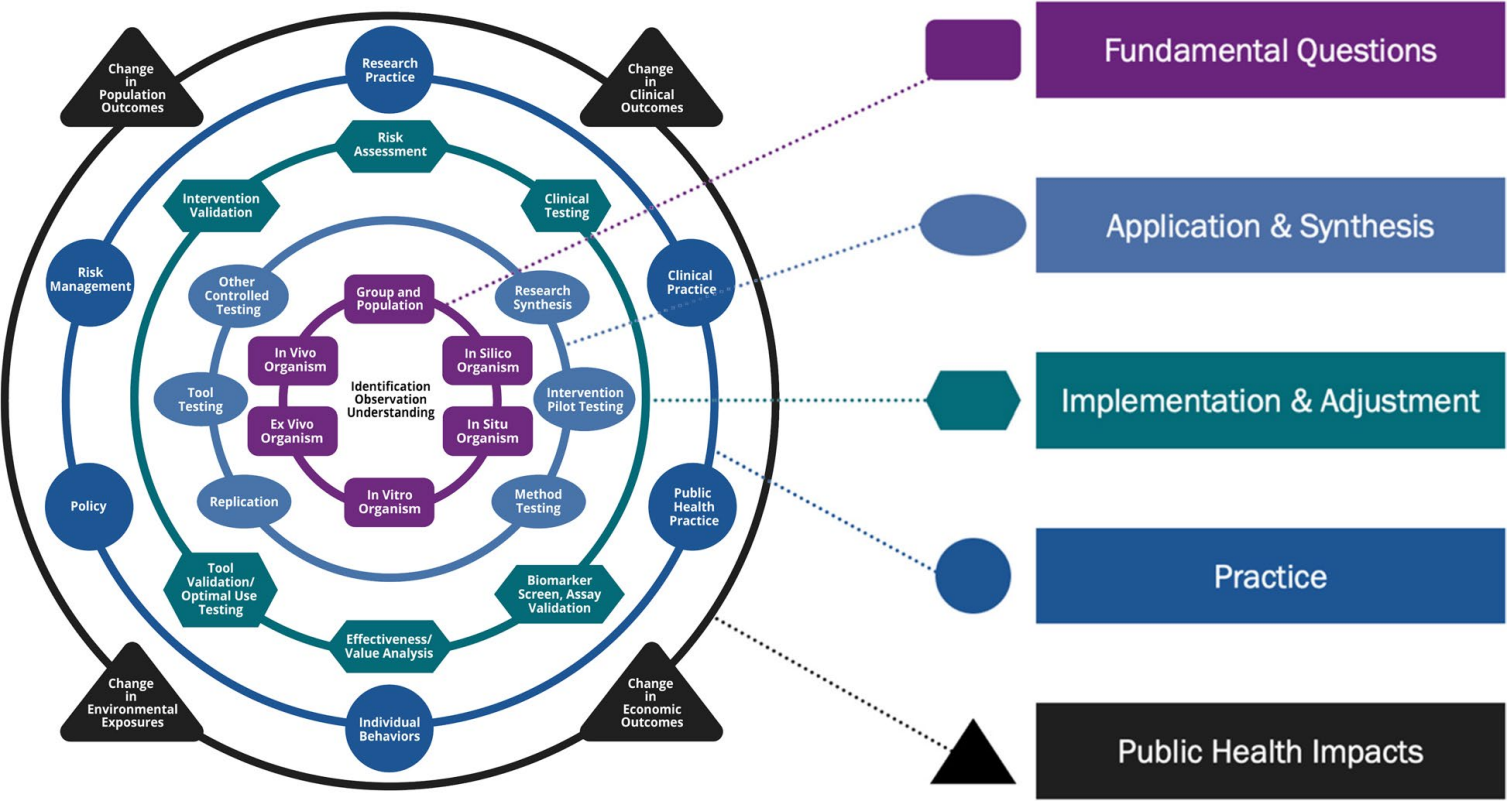
The National Institute of Environmental Health Sciences translational research framework (Reproduced from Environmental Health Perspectives with permission from the authors)

An assessment of translational impacts (RQs 2 and 3) will be captured by mapping research to recognized translational research frameworks (the National Institute of Environmental Health Sciences Translational Research Framework for Environmental Health Sciences [4], the Tufts Clinical and Translational Science Institute Six Types of Evidence Framework [5], and the NIOSH Research to Care (RTC) model [6], a logic diagram designed to help in program planning. The National Institute of Environmental Health Sciences (NIEHS) framework is especially well suited to evaluating the translation of research in an environmental disaster. This framework includes a tool for visualizing how ideas and knowledge move from the earliest stages of fundamental questioning to the later stages of impact (Fig. 1). The visualization depicts a series of concentric rings moving from research that addresses Fundamental Questions (purple ring, rectangles) to research that addresses Application and Synthesis (light blue ring, ovals). Other research includes Implementation and Adjustment (green ring, hexagons), Practice (dark blue ring, circles), and Impact (black ring, triangles). On each ring are nodes that identify approaches, methods, and activities that are typically used within each type of research.

### Reporting and visualization of results

The inventory of research (RQ1) will be reported in tables and accompanying narrative. We will present information in sequence for each item in the PICOTSS and PECOTTS frameworks. Items pertaining to the translational aspects of research (RQs 2 and 3) will be presented in tables and, where possible, we will use figures to depict concentrations of research publications along specific parts of the translational frameworks (see Fig. 1 for an example). All data collected and all data prepared for visualization and reporting will be made available with the publication of a peer reviewed and publicly available RAND Report. All RAND Reports are made avialable online and are indexed and searchable through major scientific public databases.

As this is a scoping review designed to provide data on existing research across a vast range of populations, conditions, and outcomes, we do not plan to conduct risk of bias or strength of evidence analyses at the effect level or study level. However, we will collect information (see Additional file 2) that can be used to assess risks of bias and strength of evidence in future systematic reviews on specific populations, conditions and outcomes.

## Discussion

The Program carries out an active translational research program on behalf of its more than 120,000 members. This protocol describes an approach for documenting the extent and nature of WTC translational research and its impacts. It is designed to assist the program in keeping an ongoing inventory of this research by identifying the populations, interventions, and exposures that have been studied, in what settings, and over what time frames. Beyond keeping an ongoing inventory, the Program also seeks to assess the translational impacts of WTC-related health research. This protocol is designed to address this need by mapping existing research to recognized translational frameworks and by documenting changes in translational impacts over time.

In conclusion, this work will establish a systematic process for conducting ongoing scoping reviews on the growing body of WTC-related health research. The evidence we produce from ongoing scoping reviews can be used to support Program planning and public access to research evidence.

### Supplementary Information

**Additional file 1. **Search terms.

**Additional file 2. **Query form.

**Additional file 3. **PRISMA-P (Preferred Reporting Items for Systematic review and Meta-Analysis Protocols) 2015 checklist: recommended items to address in a systematic review protocol*.

## Data Availability

Data and material will be delivered to NIOSH and made available by request. A PRISMA-P checklist was submitted to the journal with this protocol and will be made available on request.

## Abbreviations

CENTRAL: Cochrane Central Register of Controlled Trials
CTR: Clinical and translational research
NIEHS: National Institute for Environmental Health Sciences
NIOSH: National Institute of Occupational Safety and Health
PRISMA: Preferred Items for Reporting on Systematic Reviews and Meta Analyses
RTC: Research to Care
WTC: World Trade Center

## Footnotes

1 Youth are defined by the Program as individuals who were in utero or under age 18 at the time of the attacks.

2 The first update will cover research published from November 1, 2020, through December 31, 2022. Future updates will cover research published after December 31, 2022.

## References

[1] Concannon TW, Faherty LJ, Madrigano J, Mann S, Chari R, Siddiqi SM, Lee J, Hiatt L. Translational impacts of World Trade Center Health Program research: a mixed methods study. Santa Monica, CA: RAND Corporation, 2021. Available at https://www.rand.org/pubs/research_reports/RRA390-1.html. Accessed 29 Nov 2022. 10.7249/rhq-09-03-08 PMC9242573 35837518

[2] Daniels RD, Kubale T. “A way forward: the translational impacts of World Trade Center Health Program Research.” *NIOSH Science Blog,* October 26, 2021. Available at https://blogs.cdc.gov/niosh-science-blog/2021/10/26/wtchp-translational-impacts/. Accessed 29 Nov 2022.

[3] “World Trade Center Health Program Research: access 9/11 health research publications, articles, and more.” Available at https://www.cdc.gov/wtc/research.html. Accessed 29 Nov 2022.

[4] Pettibone KG, David M, Balshaw DM, Dilworth C, Christina H, Drew CH, Hall JE, Heacock M, Alfonso R, McAllister KA, O’Fallon LR, Thompson C, Walker NJ, Wolfe MS, Wright DS, Collman GW (2018). Expanding the concept of translational research: making a place for environmental health sciences. Environ Health Perspect.

[5] Selker HP, Leslie LK, Wasser JS, Plaut AG, Wilson IB, Griffith JL (2010). Tufts CTSI: comparative effectiveness research as a conceptual framework for a focus on impact. Clin Transl Sci.

[6] Reismann D. “Research-to-care model” World Trade Center Health Program; 2015 Available at: https://www.cdc.gov/wtc/pdfs/Reissman-STACJune42015.pdf. Accessed 29 Jan 2019.

[7] Kubale, Travis, Alan Katruska, Eric Pierce Brown, Robert Daniels, and Dori B. Reissman, Summary of World Trade Center Health Program Research: NIOSH Research Compendium, Washington, D.C.: National Institute for Occupational Safety and Health, December 2019. As of September 20, 2021: https://wwwn.cdc.gov/ResearchGateway/Content/pdfs/WTCHP_Research_Compendium_December_2019.pdf.

[8] Moher D, Liberati A, Tetzlaff J, Altman DG (2009). Preferred reporting items for systematic reviews and meta-analyses: the PRISMA statement. Ann Intern Med.

[9] DistillerSR: Evidence Partners; 2019. Available at https://www.evidencepartners.com/. Accessed 30 Nov 2022.

[10] Matchar DB (2012). Introduction to the methods guide for medical test reviews. J Gen Intern Med.

