# Supplementary material for: A protocol for ongoing systematic scoping reviews of World Trade Center Health research
Source: Syst Rev. 2023 Oct 10;12:193. doi: 10.1186/s13643-023-02318-x (PMC10563243; doi:10.1186/s13643-023-02318-x)
Supplement: Supplementary file 1 — Additional file 1. Search terms. [file 13643_2023_2318_MOESM1_ESM.docx]

**Statement #1**

**OVID MEDLINE**Limit to 2020-current

("world trade center" OR "world trade centre" OR wtc OR (sep* adj1"11") OR (sep* adj1"11th") OR "ground zero").ti,ab,kf.
OR

**PsycInfo (EBSCO platform)**Limit to 2020-present; phrase searching; journals

TI "world trade center" OR "world trade centre" OR AB "world trade center" OR "world trade centre" OR KW "world trade center" OR "world trade centre" OR TI "wtc" OR AB "wtc" OR KW "wtc" OR TI(sep* N1 11) OR AB(sep* N1 11) OR KW(sep* N1 11) OR TI(sep* N1 11th) OR AB(sep* N1 11th) OR KW(sep* N1 11th) OR TI "ground zero" OR AB "ground zero" OR KW "ground zero"

**CINAHL (EBSCO platform)**Limit to 2020-present; phrase searching; exclude medline records; academic journals

TI ("world trade center" OR "world trade centre") OR AB ("world trade center" OR "world trade centre") OR TI ("wtc" OR AB "wtc" OR MW ("world trade center" OR MW "world trade centre") OR TI(sep* N1 11) OR AB(sep* N1 11) OR MW(sep* N1 11) OR TI(sep* N1 11th) OR AB(sep* N1 11th) OR MW(sep* N1 11^th^) OR TI ("ground zero") OR AB ("ground zero") OR MW ("ground zero")

**Scopus (Elsevier)**Limit 2020-present; Article, Review
(notes: remove WTC from statement #1, change sep* to sept*)

TITLE-ABS-KEY("world trade center") OR TITLE-ABS-KEY("world trade centre") OR TITLE-ABS-KEY ( sept* W/0 11 ) OR TITLE-ABS-KEY ("ground zero")

AND ( EXCLUDE ( SUBJAREA , "SOCI" ) OR EXCLUDE ( SUBJAREA , "ENGI" ) OR EXCLUDE ( SUBJAREA , "ARTS" ) OR EXCLUDE ( SUBJAREA , "COMP" ) OR EXCLUDE ( SUBJAREA , "EART" ) OR EXCLUDE ( SUBJAREA , "MATE" ) OR EXCLUDE ( SUBJAREA , "PHYS" ) OR EXCLUDE ( SUBJAREA , "AGRI" ) OR EXCLUDE ( SUBJAREA , "CENG" ) OR EXCLUDE ( SUBJAREA , "ENER" ) OR EXCLUDE ( SUBJAREA , "MATH" ) OR EXCLUDE ( SUBJAREA , "CHEM" ) OR EXCLUDE ( SUBJAREA , "VETE" ) OR EXCLUDE ( SUBJAREA , "Undefined" ) ) AND ( EXCLUDE ( SRCTYPE , "Undefined" ) )

**Web of Science (Clarivate)**Limit to 2020-present
Indexes=SCI-EXPANDED, CPCI-S, BKCI-S, IC, Timespan=2020-2020
Article, Review, Early Access

(notes: remove WTC from statement #1, changed Sep* to Sept*.)

TS=("world trade center") OR TS=("world trade centre") OR TS=(sept* NEAR/0 11) OR TS=(sept* NEAR/0 11th) OR TS=("ground zero")

Exclude: RESEARCH AREAS: ( PLANT SCIENCES OR ENGINEERING OR REMOTE SENSING OR COMPUTER SCIENCE OR ASTRONOMY ASTROPHYSICS OR EDUCATION EDUCATIONAL RESEARCH OR AGRICULTURE OR ENERGY FUELS OR FOOD SCIENCE TECHNOLOGY OR CONSTRUCTION BUILDING TECHNOLOGY OR SCIENCE TECHNOLOGY OTHER TOPICS OR MATHEMATICAL METHODS IN SOCIAL SCIENCES OR NUCLEAR SCIENCE TECHNOLOGY OR CHEMISTRY OR GEOCHEMISTRY GEOPHYSICS OR THERMODYNAMICS OR PHYSICS OR METEOROLOGY ATMOSPHERIC SCIENCES OR OCEANOGRAPHY OR ROBOTICS OR MATHEMATICS OR VETERINARY SCIENCES OR MECHANICS OR SPECTROSCOPY OR GEOLOGY OR WATER RESOURCES )

**Embase (Elsevier)s**limit 2020-present; humans
article, article in press, review

("world trade center" OR "world trade centre" OR (sept* near/1 "11") OR (sept* near/1"11th") OR "ground zero"):ti,ab,kw

**Statement #2**

**OVID MEDLINE**
Limit to 2020-present

"9/11".ti,ab,kf.
AND
(terror* OR attack* OR catastroph* OR disaster* OR collaps* OR tower* OR building* OR fire* OR plane* OR airplane* OR jet* OR aircraft* OR burn* OR crash* OR hijack*).ti,ab,kf.

**PsycInfo (EBSCO platform)**Limit to 2020-present; phrase searching
Academic Journals

TI 9/11 OR AB 9/11 OR KW 9/11
AND
TI terror* OR AB terror* KW terror* OR TI attack* OR AB attack* OR KW attack* OR TI catastroph* OR AB catastroph* OR KW catastroph* OR TI disaster* OR AB disaster* OR KW disaster* OR TI collapse* OR AB collapse* OR KW collapse* OR TI tower* OR AB tower* OR KW tower* OR TI building* OR AB building* OR KW building* OR TI fire* OR AB fire* OR KW fire* OR TI plane* OR AB plane* OR KW plane* OR TI airplane* OR KW airplane* OR TI jet* OR AB jet* OR KW jet* OR TI aircraft* OR AB aircraft* OR aircraft* OR TI burn* OR AB burn* OR KW burn* OR TI crash* OR AB crash* OR KW crash* OR TI hijack* OR AB hijack* OR KW hijack*

**CINAHL (EBSCO platform)**Limit to 2020-present; phrase searching; exclude medline records
Academic Journals

TI 9/11 OR AB 9/11 OR MW 9/11
AND
TI terror* OR AB terror* OR MW terror* OR TI attack* OR AB attack* OR MW attack* OR TI catastroph* OR AB catastroph* OR MW catastroph* OR TI disaster* OR AB disaster* OR MW disaster OR TI collaps* OR AB collaps* OR MW collaps* OR TI tower* OR AB tower* OR MW tower& OR TI building* OR AB building* OR MW building* OR TI fire* OR AB fire* OR MW fire* OR TI plane* OR AB plane* OR MW plane* OR TI airplane* OR AB airplane* OR MW airplane* OR TI jet* OR AB jet* OR MW jet* OR TI aircraft* OR AB aircraft* OR MW aircraft* OR TI burn* OR AB burn* OR MW burn* OR TI crash* OR AB crash* OR MW crash* OR TI hijack* OR AB hijack* OR MW hijack*

**Scopus (Elsevier)**Limit 2020-present
(notes: remove WTC from statement #1, change sep* to sept*)

TITLE-ABS-KEY ("9/11")
AND
TITLE-ABS-KEY (terror* OR attack* OR catastroph* OR disaster* OR collaps* OR tower* OR building* OR fire* OR plane* OR airplane* OR jet* OR aircraft* OR burn* OR crash* OR hijack*) OR ABS(terror* OR attack* OR catastroph* OR disaster* OR collaps* OR tower* OR building* OR fire* OR plane* OR airplane* OR jet* OR aircraft* OR burn* OR crash* OR hijack*)

AND ( EXCLUDE ( SUBJAREA , "SOCI" ) OR EXCLUDE ( SUBJAREA , "ARTS" ) OR EXCLUDE ( SUBJAREA , "COMP" ) OR EXCLUDE ( SUBJAREA , "EART" ) OR EXCLUDE ( SUBJAREA , "PHYS" ) OR EXCLUDE ( SUBJAREA , "CHEM" ) OR EXCLUDE ( SUBJAREA , "MATE" ) OR EXCLUDE ( SUBJAREA , "CENG" ) OR EXCLUDE ( SUBJAREA , "ENER" ) OR EXCLUDE ( SUBJAREA , "AGRI" ) OR EXCLUDE ( SUBJAREA , "MATH" ) OR EXCLUDE ( SUBJAREA , "VETE" ) )

**Web of Science (Clarivate)**Limit to 2020-present
Indexes=SCI-EXPANDED, CPCI-S, BKCI-S, IC, Timespan=2001-2017

TS=("9/11")
AND
TS=(terror* OR attack* OR catastrophe* OR disaster* OR collaps* OR tower* OR building* OR fire* OR plane* OR airplane* OR jet* OR aircraft* OR burn* OR crash* OR hijack*)

**Embase (Elsevier)**limit 2020-present; humans
article, article in press, review

("9/11"):ti,ab,kw
AND
(terror* OR attack* OR catastroph* OR disaster* OR collaps* OR tower* OR building* OR fire* OR plane* OR airplane* OR jet* OR aircraft* OR burn* OR crash* OR hijack*):ti,ab,kw
