# Supplementary material for: A protocol for ongoing systematic scoping reviews of World Trade Center Health research
Source: Syst Rev. 2023 Oct 10;12:193. doi: 10.1186/s13643-023-02318-x (PMC10563243; doi:10.1186/s13643-023-02318-x)
Supplement: Supplementary file 2 — Additional file 2. Query form. [file 13643_2023_2318_MOESM2_ESM.docx]

Appendix B Query Form

| Distiller ID | Sub ID | Question | Response Options | Response option definitions |
| --- | --- | --- | --- | --- |
| Section 1: Article Identifying Information | | | | |
| 1 | | First Author (last name): |  |  |
| 2 | | First Author Primary Affiliation |  | Cut and paste the entirety of the first author's primary affiliation from the footnote. |
| 3 | | Last Author (last name) |  |  |
| 4 | | Last Author Primary Affiliation |  | Cut and paste the entirety of the last author's primary affiliation from the footnote. |
| 5 | | Keywords identified by authors (write "NA" if not included in paper) |  | Copy and paste from paper |
| 6 | | Select the funder(s) from the following list and provide the award number if available. | NIOSH [1]  NIOSH [2]  ATSDR Other CDC  NIH  CMS AHRQ  PCORI New York City Department of Health and Mental Hygiene Other public funder [name]  Other private funder [name]  Unable to assess  No funding | NIH Related Institutions:  NIH Office of the Director (OD) National Cancer Institute (NCI)  National Eye Institute (NEI)  National Heart, Lung, and Blood Institute (NHLBI)  National Human Genome Research Institute (NHGRI)  National Institute on Aging (NIA)  National Institute on Alcohol Abuse and Alcoholism (NIAAA)  National Institute of Allergy and Infectious Diseases (NIAID)  National Institute of Arthritis and Musculoskeletal and Skin Diseases (NIAMS)  National Institute of Biomedical Imaging and Bioengineering (NIBIB)  Eunice Kennedy Shriver National Institute of Child Health and Human Development (NICHD)  National Institute on Deafness and Other Communication Disorders (NIDCD)  National Institute of Dental and Craniofacial Research (NIDCR)  National Institute of Diabetes and Digestive and Kidney Diseases (NIDDK)   National Institute on Drug Abuse (NIDA)  National Institute of Environmental Health Sciences (NIEHS)  National Institute of General Medical Sciences (NIGMS)  National Institute of Mental Health (NIMH)  National Institute on Minority Health and Health Disparities (NIMHD)   National Institute of Neurological Disorders and Stroke (NINDS)  National Institute of Nursing Research (NINR)  National Library of Medicine (NLM)  NIH Clinical Center (CC)  Center for Information Technology (CIT)  Center for Scientific Review (CSR)  Fogarty International Center (FIC)  National Center for Advancing Translational Sciences (NCATS) National Center for Complementary and Integrative Health (NCCIH) |
| 7 | | Should this article have been excluded? | Yes No |  |
|  | *8* | *Reason for exclusion?* | *Not English Not research Not relevant to 9/11 Not relevant to 9/11 populations Not relevant to 9/11 conditions, care or outcomes* | Not in English;   Not research- Ask if data was collected for the purpose of answering a question. Editorials, memorials, letters to the editor, etc. are not research.  Not relevant to 9/11: a paper may mention 9/11, for instance a study of people since 9/11, but the study is not about 9/11. May be using 9/11 as an inflection point.  Not relevant to 9/11 populations- focused on 9/11 but not about the WTCHP populations directly impacted by the disaster. ; not about health or health care  Not relevant to 9/11 conditions care or outcomes: meets the above criteria but is not a health related study. |
| Section 2: Article Content | | | | |
| 9 | | Copy and paste the full sentence(s) in which the objectives of the study are stated [typically found in the last paragraph of the Introduction or Background section]. |  | We prefer full text from the main body of the articles, the Introduction or Background section, rather than from the abstract. |
| 10 | | What type of study is this? | Exposure Characterization Exposure-Outcome Linkage Outcome Modification | This question is focused on the objectives of the study, what is the point of the study? |
| 11 | | According to the Tufts CTSI 6 types of evidence framework, does the study... [choose one] |  |  |
| ∙ | | *Set priorities for future research (evidence prioritization)* |  | Evidence prioritization occurs before much of what we generally think of as "research." It's aim is to determine what kind of evidence needs updating. |
| ∙ | | *Develop new evidence about an intervention or exposure [evidence generation]?* |  |  |
|  | *12* | *Were the study participants randomly sampled from the study population?* | *Yes No* | Random sampling is based on how the study data is brought into the analytic file for the study, if this is done in a random way. In other words, random sampling from a population involves some procedure to ensure members of the population have an equal or predicted probability of being included in the study. |
|  | *13* | *Describe the sampling procedure* |  |  |
|  | *14* | *Were study data collected prospectively?* | *Yes No* | Prospective includes primary data collected over the course of the study: prospective data is data that is not complete and has not been collected at the start of the study. If the data already exists, then that is retrospective (example: a paper that solely uses registry data). A study often combines the two, if it is combined then choose prospective.  This is about data collection.  It can be unclear if data was prospectively collected if a study solely using Registry data is conducted by Registry authors (we wouldn't know if they had collected the Registry data having begun the study or with this particular study in mind). In these situations, choose retrospective unless the authors say they prospectively collected data (different from simply stating that the WTCHR is a prospective cohort). |
|  | *15* | *Choose all that apply.* | *Exposure(s) Intervention(s) Outcome(s)* |  |
|  | *16* | *Were any study data measured longitudinally?* | *Yes- at least some study data were longitudinal No- all study data were cross-sectional* | Cross-sectional studies use observations of subjects from a single point in time. Longitudinal studies use observations of subjects over a period of time. |
|  | *17* | *Were outcome(s) of interest measured at baseline?* | *Yes No* | Baseline outcome measures are collected before the participant is exposed or treatment is assigned  If a subject is determined as having a “positive” outcome before exposure (say, PTSD before 9/11), and they are excluded, that is not considered a baseline measurement.  SKIP PATTERN: only for longitudinal |
|  | *18* | *Were outcome(s) of interest measured more than once after the intervention or exposure was first measured?* | *Yes No* | Outcomes repeated post-intervention or post-exposure are collected more than once after the intervention or exposure is recorded.   SKIP PATTERN: only for longitudinal |
|  | *19* | *What kind of data were used to ascertain exposure(s) (for exposure studies) or intervention(s) (for intervention studies)? (check all that apply)* | *Administrative data Clinical data Research data Population registry data Environmental monitoring data Other* | *This questions asks you to identify the purpose for which the data were originally collected.   Ascertainment: this is the role of the analysis team in determining if the person had been exposed or received the intervention.*  Administrative data are those that were originally collected for administrative purposes, such as for making insurance claims.  Clinical data are those that were originally collected for clinical management purposes, such as the results of laboratory, imaging, and function testing.   Research data are collected for the purpose of conducting research, such as the data that are recorded in a clinical trial.   Population registry data are collected to support or monitor the health of a specified population. Please select either “WTC Health Registry data” or “Other registry data.”   Environmental monitoring data are collected to monitor an environment.  Data about an intervention: The intervention is recorded in a researcher’s analytic dataset. We are asking what kind of data this record originated from. For instance • If researchers looked in claims to find patients who received a certain procedure, you would answer “administrative data” • If researchers looked in an electronic health record for the procedure, you would answer “clinical data” • If the intervention was applied as part of a research program or experiment, you would answer “research data” • If a record of the procedure was taken from a registry, answer “population registry data” • It is unlikely that an intervention is applied and recorded as part of an environmental monitoring program but not inconceivable. Answer “environmental monitoring data” |
|  | *20* | *Clinical Data: Were diagnostic tests (e.g. laboratory tests or spirometry) performed to ascertain exposure(s) or confirm intervention(s))?* | *Yes No* | Note: We no longer consider validated scales an example of a diagnostic test. Please choose no in the case of validated scales. |
|  | *21* | *Research Data: Were diagnostic tests (e.g. laboratory tests or spirometry) performed to ascertain exposure(s) or confirm intervention(s))?* | *Yes No* |  |
|  | *22* | *Population Registry data* | *WTCHP Registry Other registry* | Unless stated otherwise, assume WTCHP registry if discussing an unspecified registry and WTC populations.  Registry populations: https://www1.nyc.gov/site/911health/about/who-is-enrolled.page |
|  | *23* | *Please select all the apply:* | *WTCHP Registry Wave 1 WTCHP Registry Wave 2 WTCHP Registry Wave 3 WTCHP Registry Wave 4 Unable to assess* |  |
|  | *24* | *What kind of data were used to ascertain outcomes in this study? (check all that apply)* | *Administrative data Clinical data Research data Population registry data Environmental monitoring data Other* | See above. |
|  | *25* | *Clinical Data: Were diagnostic tests (e.g. laboratory tests or spirometry) performed to ascertain exposure(s) or confirm intervention(s))?* | *Yes No* |  |
|  | *26* | *Research data: Were diagnostic tests (e.g. laboratory tests or spirometry) performed to ascertain exposure(s) or confirm intervention(s))?* | *Yes No* |  |
|  | *27* | *Population Registry data* | *WTCHP Registry Other registry* |  |
|  | *28* | *Please select all the apply:* | *WTCHP Registry Wave 1 WTCHP Registry Wave 2 WTCHP Registry Wave 3 WTCHP Registry Wave 4 Unable to assess* |  |
|  | *29* | *Did the study include an external comparison group (i.e. control group)?* | *Yes No* | This asks for the main comparison made in the paper, whether that be to control, exposed or not exposed, etc. |
|  | *30* | *Were study participants randomly assigned to different comparison groups?* | *Yes No* |  |
| ∙ | | *Synthesize or review other studies (evidence synthesis)* |  | Evidence syntheses combine the results from prior studies into a synthesis to describe a body of literature or to produce a summary effect estimate. There are several types of evidence synthesis (see descriptions and examples to the right of each response option). |
|  | 31 |  | Narrative Review Systematic Review Meta-analysis Network Meta-analysis Other | A Narrative Review describes or discusses the state of science of a specific topic or theme from a theoretical and contextual point of view, but is not based on systematic search, inclusion, and abstraction procedures. In other words: an expert doing a review without a formal review of the literature A Systematic Review uses systematic methods to collect secondary data, critically appraise research studies, and synthesize (but not combine) them. A Meta-analysis is one kind of systematic review; it is a statistical analysis combining results of multiple scientific studies. A Network Meta-analysis is a meta-analysis in which multiple treatments (that is, three or more) are being compared using both direct comparisons of interventions within randomized controlled trials and indirect comparisons across trials based on a common comparator. |
| ∙ | | *Integrate evidence from other studies in decision models, predictive models, or other models (evidence interpretation and integration)?* |  | Evidence interpretation and integration uses estimates from previous studies in simulations to extend the findings to new questions |
|  | *32* |  | *Clinical decision modeling Health modeling Economic modeling Exposure modeling Other* | Clinical decision modeling uses simulated decisions and probabilities to estimate the expected outcomes of a clinical decision problem.  Health modeling characterizes the health impact of a condition, treatment, intervention, or a health state on a population.   Economic modeling characterizes the economic impact or an intervention, medical technology, or a health state on a population.  Exposure assessment modeling characterizes the nature and extent of an environmental or experiential exposure on a population. Other |
| ∙ | | *Generate evidence about implementation of a program or policy (dissemination and application)?* |  |  |
| ∙ | | *Evaluate other research (feedback and assessment)?* |  |  |
| *33* | | *Was exposure ascertained retrospectively?* | *Yes No* | Retrospective exposure assessment occurs when historical exposure is ascertained once the outcome is known.  When it is possible that the occurrence of disease could influence the recording of exposure, this is considered ‘retrospective’. This is about exposure ascertainment.   If there's simultaneous prospective data collection (question 14) of exposure and outcome (e.g. interview or survey asking about both after study start), it is unclear whether the exposure was ascertained after the outcome was known. In those situations, we'll say exposure is retrospectively ascertained.   If there are multiple exposures and some where determined retrospectively while some were determined prospectively, answer no. |
| 34 | | What type of study exposure(s) is/are used in the study? | 9/11 related chemical hazard Other 9/11 related hazard Other health conditions Other | Remember exposure can mean two different things here: the main study exposure to a predictor of an outcome, and exposure to 9/11 as an "main study exposure"   Only include the main or other important (but not main) exposures--do not include covariates.   Age, gender, other demographic variables are not exposures even if they are specifically mentioned as being examined in the objective. They would either be covariates or part of the subgroup analysis.  The exposures in some papers may use composite measures which include other components including some that are listed or that use measures that don't exactly align with the listed exposures. In those cases, check the listed components and then also check other and name the composite/other measure, specifying that it is composite in some way or that it measures something else in addition to the listed terms. |
|  | *35* | *9/11 related chemical hazard* | *Group 1: Persistent organic pollutants, such as PCDDs, PBDDs, PCDFs, PCNs, and PCBs. Group 2: PAHs or other organic compounds not in Group 1 (other VOCs, benzene) Group 3: metals (aluminum, cadmium, chromium, iron, lead, nickel, or titanium) Group 4: other inorganics, such as radionuclides, silica, or asbestos  Group 5: Gross particulate matter (PM) such as dust, glass fragments, quarts grains, wood fibers, or other general debris Group 6: any other agent not listed in Group 1-5.(specify)* | Please only choose Groups 1-5 if a significant portion of the group is specifically mentioned in the paper. Otherwise, choose group 6 and specify in the free text comment.   This is only for 9/11 related chemical hazard exposure. |
|  | *36* | *Other 9/11 related hazard* | *Cold stress Heat stress Musculoskeletal injury from fall or event Bloodborne pathogens Present in WTC building on 9/11; no other physical or biological hazard noted Caught in dust cloud on 9/11 Present in WTC building Present in NYC WTC area  Present in Shanksville  Present at the Pentagon  PTSD Other (free text)* | This is only for 9/11 related physical, biological or other hazard exposure  If the condition appears on the list of 9/11 conditions then include under "Other 9/11 related hazard" unless it’s clear that it isn’t 9/11 related--in which case include under "Other health conditions". For example if a paper studies both what it calls "9/11 related PTSD" and "MDD" (notice that they haven't stated explicitly that MDD is 9/11 related), then under "Other 9/11 related hazard," check PTSD and check other, including MDD free response. |
|  | *37* | *Other health conditions* | *Physical condition  Neuropsychiatric condition* | Health condition exposure may or may not be 9/11 related |
|  | *∙* | *Other* |  |  |
| 38 | | Which categories of exposure intensity were measured? [*Select all that apply]* | Arrival date Duration at WTC site Exposed to 9/11 dust cloud Any work on the pile Unable to assess/Not applicable | SKIP PATTERN: Must have selected 9/11 related physical hazard or other 9/11 related hazard above.  "Pile" refers to the WTC ruins. |
| 39 | | What type of intervention(s) or treatment(s) is/are used in the study? | Screening or testing (specify) Treatment or clinical management (specify) Other |  |
| 40 | | What type of comparator is used in the study? (choose one) |  | When a comparator is used, the total study population is divided into two or more analytic groups: group 1 is the intervention/exposed group while group(s) 2+: is(are) the comparator group(s).   SKIP PATTERN: only if answered Yes to 29. is there a comparison group.A50:C52 |
|  | 41 | *If intervention study (select all that apply)* | *Other treatment  Same treatment, other dose, or level or duration of intervention  Usual care  Placebo* | Usual care: only select if terms like "usual care," 'care as usual," or "standard of care," otherwise select another treatment. |
|  | 42 | *If exposure study (select all that apply)* | *Unexposed Another level or duration of exposure* | “Exposure study” is used to refer to observational epidemiology studies throughout this form. |
| 43 | | Total duration of time that subjects received the intervention. | Up to 1 day Up to 1 week Up to 1 month Up to 3 months Up to 6 months More than 6 months (specify) Unable to assess Multiple interventions (specify) | We are interested in knowing how long someone was exposed to the intervention.  Select the maximum duration of time for multiple interventions. If you can only answer for some of the multiple , select the Multiple interventions answer and fill in the details for what you can and cannot assess. If there are multiple interventions and all are unable to assess, just choose unable to assess.  When selecting more than 6 months, please specify the exact duration of time. 26 weeks is 6 months. |
|  | *44* | *Please fill in the exact measurement of time.* |  |  |
|  | *45* | *Please select the unit of time used.* | *Hour(s) Day(s) Month(s) Year(s)* |  |
| 46 | | Total duration of time that subjects were exposed. | Up to 1 day Up to 1 week Up to 1 month Up to 3 months Up to 6 months More than 6 months (specify) Unable to assess Multiple exposures (specify) | We are interested in knowing how long someone was exposed.  Select the maximum duration of time for multiple exposures. If you can only answer for some of the multiple exposures, select the Multiple exposures answer and fill in the details of what you can and cannot assess. If there are multiple exposures and all are unable to assess, just choose unable to assess.  When selecting more than 6 months, please specify the exact duration of time. |
|  | *47* | *Please fill in the exact unit of time.* |  |  |
|  | *48* | *Please select the measure of time used.* | *Hour(s) Day(s) Month(s) Year(s)* |  |
| 49 | | Specify all study outcomes. | Covered conditions  Non-covered conditions | Covered conditions fall into 4 categories: acute traumatic injury, aerodigestive disorders, cancer, and neuropsychiatric health disorders.  Non-covered conditions include: stroke, irritable bowel syndrome, cardiovascular disease (hypertension, atherosclerosis), dementia, Parkinson’s disease and Parkinsonism, Neuropathy, Autoimmune conditions (rheumatoid arthritis, multiple sclerosis, lupus, encephalitis),   Include as a 9/11 condition unless it is clear that it is not 9/11 related. |
|  | 50 | Select all that apply: | Physical health-covered conditions Neuropsychiatric health- covered conditions |  |
|  | 51 | *Physical Health- covered conditions (select all that apply)* | *Acute Traumatic Injury Aerodigestive Disorders Cancer Musculoskeletal Disorders* | Acute traumatic injury: Burn, Complex sprain, Eye injury, Fracture, Head trauma, Other similar acute traumatic injuries, Tendon tear  Musculoskeletal Disorders: Carpal tunnel syndrome (CTS), Low back pain, Other musculoskeletal disorders |
|  | 52 | *Acute Traumatic Injury* | *Burn Complex sprain Eye injury Fracture Head trauma Other similar acute traumatic injuries Tendon tear* |  |
|  | 53 | *Burn: Which option best describes this outcome?* | *Primary Secondary Not Specified* |  |
|  | 54 | *Complex sprain* | *Primary Secondary Not Specified* |  |
|  | 55 | *Eye injury: Which option best describes this outcome?* | *Primary Secondary Not Specified* |  |
|  | 56 | *Fracture: Which option best describes this outcome?* | *Primary Secondary Not Specified* |  |
|  | 57 | *Head trauma: Which option best describes this outcome?* | *Primary Secondary Not Specified* |  |
|  | 58 | *Other similar acute traumatic injuries: Which option best describes this outcome?* | *Primary Secondary Not Specified* |  |
|  | 59 | *Tendon tear: Which option best describes this outcome?* | *Primary Secondary Not Specified* |  |
|  | 60 | *Aerodigestive disorders* | *Asthma Chronic cough syndrome Chronic laryngitis Chronic nasopharyngitis Chronic respiratory disorder due to fumes/vapors Chronic rhinosinusitis Gastroesophageal reflux disorder (GERD) Interstitial lung disease (e.g. sarcoidosis) Reactive airways dysfunction syndrome (RADS) Sleep apnea exacerbated by or related to another condition in the list of aerodigestive disorders Upper airway hyperreactivity WTC-exacerbated chronic obstructive pulmonary disease (COPD)* |  |
|  | 61 | *Asthma: Which option best describes this outcome?* | *Primary Secondary Not Specified* |  |
|  | 62 | *Chronic cough syndrome* | *Primary Secondary Not Specified* |  |
|  | 63 | *Chronic laryngitis: Which option best describes this outcome?* | *Primary Secondary Not Specified* |  |
|  | 64 | *Chronic nasopharyngitis: Which option best describes this outcome?* | *Primary Secondary Not Specified* |  |
|  | 65 | *Chronic respiratory disorder due to fumes/vapor: Which option best describes this outcome?* | *Primary Secondary Not Specified* |  |
|  | 66 | *Chronic rhinosinusitis: Which option best describes this outcome?* | *Primary Secondary Not Specified* |  |
|  | 67 | *Gastroesophageal reflux disorder (GERD): Which option best describes this outcome?* | *Primary Secondary Not Specified* |  |
|  | 68 | *Interstitial lung disease (e.g. sarcoidosis): Which option best describes this outcome?* | *Primary Secondary Not Specified* |  |
|  | 69 | *Reactive airways dysfunction syndrome (RAD): Which option best describes this outcome?* | *Primary Secondary Not Specified* |  |
|  | 70 | *Sleep apnea exacerbated by or related to another condition in the list of aerodigestive disorders: Which option best describes this outcome?* | *Primary Secondary Not Specified* |  |
|  | 71 | *Upper airway hyperreactivity: Which option best describes this outcome?* | *Primary Secondary Not Specified* |  |
|  | 72 | *WTC-exacerbated chronic obstructive pulmonary disease (COPD): Which option best describes this outcome?* | *Primary Secondary Not Specified* |  |
|  | 73 | *Cancer* | *Any Cancer (cancer type not specified)  Blood and lymphoid tissue Digestive System Eye and orbit Female breast Female reproductive organs Head and neck Respiratory system Skin (Melanoma and non-Melanoma) Soft tissue Thyroid Urinary system Mesothelioma Other rare cancers* |  |
|  | 74 | Specify from bullet list: |  | Diffuse non-Hodgkin lymphoma Follicular (nodular) non-Hodgkin lymphoma Hodgkin's disease Leukemia of unspecified cell type Lymphoid leukemia Malignant immunoproliferative diseases Monocytic leukemia Acute monocytic leukemia Chronic monocytic leukemia Monocytic leukemia, unspecified Multiple myeloma and malignant plasma cell neoplasms Myeloid leukemia Other and unspecified lymphoid, hematopoietic, and related tissue Other and unspecified types of non-Hodgkin lymphoma Other leukemias of specified cell type Peripheral and cutaneous T-cell lymphoma |
|  | 75 | Blood and lymphoid tissue: Which option best describes this outcome? | *Primary Secondary Not Specified* |  |
|  | 76 | Specify from bullet list: |  | Colon Esophagus Liver and intrahepatic bile ducts Other and ill-defined digestive organs Rectosigmoid junction Rectum Retroperitoneum and peritoneum Stomach |
|  | 77 | Digestive System: Which option best describes this outcome? | *Primary Secondary Not Specified* |  |
|  | 78 | Specify from bullet list: |  | Eye and adnexa |
|  | 79 | Eye and orbit: Which option best describes this outcome? | *Primary Secondary Not Specified* |  |
|  | 80 | Specify from bullet list: |  | Breast |
|  | 81 | Female breast: Which option best describes this outcome? | *Primary Secondary Not Specified* |  |
|  | 82 | Specify from bullet list: |  | Ovary |
|  | 83 | Female reproductive organs: Which option best describes this outcome? | *Primary Secondary Not Specified* |  |
|  | 84 | Specify from bullet list: |  | Accessory sinuses Base of tongue Floor of mouth Gum Hypopharynx Larynx Lip Nasopharynx Other and ill-defined conditions in the lip, oral cavity, and pharynx Other and unspecified major salivary glands Other and unspecified part of the mouth Other and unspecified parts of the tongue Oropharynx Palate Parotid gland Piriform sinus Tonsil |
|  | 85 | Head and neck: Which option best describes this outcome? | *Primary Secondary Not Specified* |  |
|  | 86 | Specify from bullet list: |  | Bronchus and lung Heart, mediastinum, and pleura Other and ill-defined sites in the respiratory system and intrathoracic organs |
|  | 87 | Respiratory system: Which option best describes this outcome? | *Primary Secondary Not Specified* |  |
|  | 88 | Specify from bullet list: |  | Malignant melanoma of skin Other malignant neoplasms of skin |
|  | 89 | Skin (Melanoma and non-Melanoma) : Which option best describes this outcome? | *Primary Secondary Not Specified* |  |
|  | 90 | Specify from bullet list: |  | Other connective and soft tissue Peripheral nerves and autonomic nervous system |
|  | 91 | Soft tissue: Which option best describes this outcome? | *Primary Secondary Not Specified* |  |
|  | 92 | Specify from bullet list: |  | Thyroid gland |
|  | 93 | Thyroid: Which option best describes this outcome? | *Primary Secondary Not Specified* |  |
|  | 94 | Specify from bullet list: |  | Bladder Kidney Other and unspecified urinary organs Prostate Renal pelvis Ureter |
|  | 95 | Urinary system: Which option best describes this outcome? | *Primary Secondary Not Specified* |  |
|  | 96 | Specify from bullet list: |  | Mesothelioma |
|  | 97 | Mesothelioma: Which option best describes this outcome? | *Primary Secondary Not Specified* |  |
|  | 98 | Specify from bullet list: |  | Malignant neoplasms of the— • adrenal gland and other endocrine glands and related structures • anus and anal canal • bone and articular cartilage • breast among men • gallbladder and other parts of biliary tract • meninges, brain, spinal cord, cranial nerves, and other parts of central nervous system • pancreas • penis and testis • placenta • small intestine • thymus • vulva, vagina, and cervix uteri (invasive only) Malignant neuroendocrine neoplasm, including carcinoid tumors Myeloid neoplasms, including myelodysplastic syndromes, myeloproliferative neoplasms, myelodysplastic/myeloproliferative neoplasms, and myeloid malignancies associated with eosinophilia and abnormalities of growth factor receptors derived from platelets or fibroblasts |
|  | 99 | Other rare cancers: Which option best describes this outcome? | *Primary Secondary Not Specified* |  |
|  | 100 | *Musculoskeletal Disorders* | *Carpal tunnel syndrome (CTS) Low back pain Other musculoskeletal disorders* |  |
|  | 101 | *Carpal tunnel syndrome (CTS): Which option best describes this outcome?* | *Primary Secondary Not Specified* |  |
|  | 102 | *Low back pain: Which option best describes this outcome?* | *Primary Secondary Not Specified* |  |
|  | 103 | *Other musculoskeletal disorders: Which option best describes this outcome?* | *Primary Secondary Not Specified* |  |
|  | 104 | *Neuropsychiatric health – covered conditions* | *Acute stress disorder Adjustment disorder Anxiety disorder (NOS) Depression (NOS) Major depressive disorder Dysthymic disorder Generalized anxiety disorder Panic disorder Post-traumatic stress disorder (PTSD) Alcohol or substance use disorder* |  |
|  | 105 | *Acute stress disorder: Which option best describes this outcome?* | *Primary Secondary Not Specified* |  |
|  | 106 | *Adjustment disorder: Which option best describes this outcome?* | *Primary Secondary Not Specified* |  |
|  | 107 | *Anxiety disorder (NOS): Which option best describes this outcome?* | *Primary Secondary Not Specified* |  |
|  | 108 | *Depression (NOS): Which option best describes this outcome?* | *Primary Secondary Not Specified* |  |
|  | 109 | *Dysthymic disorder: Which option best describes this outcome?* | *Primary Secondary Not Specified* |  |
|  | 110 | *Generalized anxiety disorder: Which option best describes this outcome?* | *Primary Secondary Not Specified* |  |
|  | 111 | *Major depressive disorder: Which option best describes this outcome?* | *Primary Secondary Not Specified* |  |
|  | 112 | *Panic disorder: Which option best describes this outcome?* | *Primary Secondary Not Specified* |  |
|  | 113 | *Post-traumatic stress disorder (PTSD): Which option best describes this outcome?* | *Primary Secondary Not Specified* |  |
|  | 114 | *Alcohol or substance use disorder: Which option best describes this outcome?* | *Primary Secondary Not Specified* |  |
|  | 115 | *Non-covered conditions (select all that apply)* | *Physical Health Neuropsychiatric Health Other Non-covered Primary Outcomes Other Non-covered secondary outcomes* |  |
|  | 116 | *Physical Health* | *Stroke Irritable bowel syndrome Cardiovascular diseases (hypertension, atherosclerosis) Neuropathy Autoimmune conditions (rheumatoid arthritis, multiple sclerosis, lupus, encephalitis) Biliary cirrhosis Other physical conditions* |  |
|  | 117 | *Stroke: Which option best describes this outcome?* | *Primary Secondary Not Specified* |  |
|  | 118 | *Irritable bowel syndrome: Which option best describes this outcome?* | *Primary Secondary Not Specified* |  |
|  | 119 | *Cardiovascular diseases (hypertension, atherosclerosis): Which option best describes this outcome?* | *Primary Secondary Not Specified* |  |
|  | 120 | *Neuropathy: Which option best describes this outcome?* | *Primary Secondary Not Specified* |  |
|  | 121 | *Autoimmune conditions: (rheumatoid arthritis, multiple sclerosis, lupus, encephalitis): Which option best describes this outcome?* | *Primary Secondary Not Specified* |  |
|  | 122 | *Biliary cirrhosis: Which option best describes this outcome?* | *Primary Secondary Not Specified* |  |
|  | 123 | *Other physical conditions (free text)* |  |  |
|  | 124 | *Which option best describes this outcome?* | *Primary Secondary Not Specified* |  |
|  | 125 | *Neuropsychiatric Health* | *Dementia Parkinson's disease and Parkinsonism Other Neuropsychiatric conditions* |  |
|  | 126 | *Dementia: Which option best describes this outcome?* | *Primary Secondary Not Specified* |  |
|  | 127 | *Parkinson's disease and Parkinsonism: Which option best describes this outcome?* | *Primary Secondary Not Specified* |  |
|  | 128 | *Other neuropsychiatric conditions (free text)* |  |  |
|  | 129 | *Which option best describes this outcome?* | *Primary Secondary Not Specified* |  |
|  | 130 | *Other Non-covered Primary Outcome- free text* |  |  |
|  | 131 | *Other Non-covered secondary outcomes- Free Text* |  |  |
| All Outcomes-related questions following this break refer to the Primary outcome(s) only. | | | | |
| 132 | | How are the primary study outcome(s) measured or assessed? | Self report Clinical judgement without diagnostic testing Clinical judgement with diagnostic testing (specify) | Here we are not looking for the data source but rather the actual measure or assessment tool or laboratory or other test that was used.  Diagnostic testing refers to laboratory testing, pulmonary function tests, imaging such as chest x-rays and MRIs, biopsies, and other objective measurement that goes beyond the clinical judgment   validated scales used to measure/diagnosis depression, PTSD, etc. like the PHQ-9 and PCL-17, are considered self report validated scales |
|  | *133* | *Self-report: Were validated scales used?* | *Yes (specify) No* | *Please type the scale used in the paper.* |
|  | *134* | *Clinical judgement without diagnostic testing: were validated scales used?* | *Yes (specify) No* | *Please type the scale used in the paper.* |
| 135 | | Duration of follow-up: | Up to one day Up to 1 week Up to 1 month Up to 3 months Up to 6 months More than 6 months (specify) Unable to assess Not applicable | Choose the longest possible duration.   Follow-up is the length of time from intervention or exposure to final outcome measurement.   Select the longest duration of follow-up time for multiple outcomes. If unknown, select unable to assess. |
|  | 136 | Please fill in the exact unit of time. |  |  |
|  | 137 | Please select the measure of time used. | Hour(s) Day(s) Month(s) Year(s) |  |
| 138 | | What category best describes the population under study? [population] (select all that apply) | Human studies: Group or Population Non-human animal studies: In vivo organism In vitro In silico Other | In vivo: living organisms or parts of organisms  In vitro: in a test tube/laboratory  In silico: computer simulation |
| ∙ | | *Group or Population* |  |  |
|  | 139 | Which type of group or population? | Infants/children/adolescents (<18)  Adults (>18) |  |
|  | 140 | *Select all that apply:  Born before 9/11 and exposed on 9/11 Born before 9/11 and parent was exposed on 9/11 Was in utero on 9/11 and parent was exposed on 9/11* |  |  |
| ∙ | | *Adults* |  | Only mark the following if the study specifically addresses them as a study objective OR describes in inclusion/exclusion criteria |
|  | 141 | Was the study exclusively about adults of a specific age group? | Yes No | Look at the objectives OR inclusion/exclusion criteria. Check yes if the study is exclusively about adults of certain ages. This question is different from question 148 on subgroup analysis: this question is asking about whether the entire analysis, the paper itself, is about a particular age range. Q148 asks about whether the study drills down on differences by age within the larger study population. |
|  | 142 | Select the included age group. | Adults, non-elderly (>18<=65) Adults, elderly (>65) None of the above |  |
|  | 143 | Was the study exclusively about a subpopulation of adults? | Yes No | Look at the objectives OR inclusion/exclusion criteria. Check yes if the study is exclusively about adults of a specific gender or race or other defining demographic characteristic. This question is different from question 148 on subgroup analysis: this question is asking about whether the entire analysis--the paper itself--is about a particular group of people. Q148 asks about whether the study drills down on differences by subgroup type within the larger study population. (I've removed language about including adults of a specific gender who were the subject of an explicit subgroup analysis). |
|  | 144 | Select the included subpopulation(s). | Women Pregnant women Men None of the above |  |
| *145* | | *Select all WTCHP population categories studied.* |  | Details on the WTCHP populations: https://www1.nyc.gov/site/911health/about/who-is-enrolled.page |
|  | *∙* | *WTC responder* |  |  |
|  | *146* | *Select all that apply:  NYC responders Pentagon Responders Shanksville, PA responders* |  |  |
|  | *147* | *Select all that apply:  FDNY and related personnel General responders (e.g. law enforcement: WTC rescue, recovery and cleanup workers)* |  | General responders includes both uniformed and non-uniformed persons.  WTCHR includes firefighters unless otherwise specified. |
|  | *∙* | *WTC survivor* |  |  |
|  | *∙* | *Other (free text)* |  |  |
| 148 | | Do the study objectives describe a specific subgroup analysis (i.e., disparities in access, quality or outcomes by subgroup)? If yes, specify which subgroup. | Race/ethnicity Age Gender Other  No Not applicable | Look at the objectives and the tables. Look at objectives to see if these demographic variables are being examined in a focused manner (not simply along the lines of, "we'll be examining demographic correlates"; any results here would be found incidentally). If not specifically mentioned in objectives or if mentioned in the aforementioned exploratory way, then look at the tables--demographic factors would occupy columns (not rows) or their ORs would be adjusted by other covariates. If neither the objectives or tables suggests that the demographic variables were used for subgroup analysis, most likely covariates. |
| 149 | | What is the number of observations in the primary study population? | Free text [do not use commas to separate numerals] Not reported Not applicable | Include everyone who was included in the study, combine if there is a control and test group. |
| 150 | | Setting Type: | Inpatient Ambulatory/outpatient Emergency department Post-acute care or skilled nursing facility Laboratory Community (*describe)* Other *(describe)* | Ambulatory setting includes health clinics, outpatient facilities, and doctor’s offices. If intervention is provided by behavioral or mental health providers also choose this option.   Laboratory setting includes diagnostic, clinical or research laboratories  Hospital setting refers to an inpatient setting.  Community setting includes studies where the population sample is drawn from community residents (N.B., most exposure studies fall here) taken outside of a clinical intervention setting. |
| 151 | | WTCHP settings (check if study specifically mentions that it was conducted in one of the following): | WTCHP Nationwide Provider Network (NPN)  WTCHP Clinical Centers of Excellence (CCE) Not applicable/None of the above | If you think you are about to "not applicable," please do a google search on your setting name to confirm that they do not have any relationships to the CCE list.   Use this rule most of the time: If any one of the authors is from the School of Medicine associated with a CCOE (doesn’t have to be the same department specified in the CCOE list) AND the paper describes the study being conducted in a clinic, the study should be listed as a CCoE study. However, if you can find strong evidence in a paper that has a CCOE affiliation that the study wasn't conducted at a CCOE (say, they state that the study was conducted in a non-CCOE clinic specifically), then mark as NA. |
| Section 3: Study Findings and Translation | | | | |
| 152 | | What are the study’s main findings? |  | Copy and paste the full sentence(s) from Conclusions of structured abstract (or find the study conclusions if no structured Conclusions section), or from first paragraph of Discussion |
| 153 | | What are the study' covariates (other variables) that were included in the statistical model for confounding adjustment? |  | Copy and paste the full sentence(s) from the Methods, or more specifically, Statistical Analysis, section of the paper. Also search tables and language in the paper for "confounding," "controlling," "adjusting" to find covariates and include those as well.  Skip Pattern: Only shows up if answer to 29 is "no." |
| 154 | | What are the study's stated limitations? |  | Copy and paste the full sentence(s) from Conclusions section that discusses limitations |
| 155 | | What are the study's stated implications for future research, practice or policy? |  | Copy and past the full sentence(s), typically found in the last paragraphs of the paper |
| 156 | | Were stakeholders reported as being involved in the research? | Yes No | Stakeholders in research are individuals or organization who are responsible or affected by health-related decisions that can be informed by research evidence |
|  | *157* | *Which type of stakeholders were involved in the research?* | *Survivors Responders Clinical Providers Non-clinical Caregivers Policymakers Family members Advocates* | Stakeholder roles in research may include, but are not limited to, co-author, advisor, and reviewer. Reporting about stakeholders as the subjects of research (e.g., the views of survivors on the list of covered conditions), however, does not count as engagement in research. |
| 158 | | NIH Translational Framework |  | Work down from T4 to T0. When one fits the paper, select that and stop. |
|  | *159* | *T4 Research: Translation to communities* | *Population level outcomes research Monitoring of morbidity, mortality, benefits, and risks Impacts of policy and change* |  |
|  | *160* | *T3 Research: Translation to practice* | *comparative effectiveness research  post-marketing studies Clinical outcomes research Health services research Dissemination & implementation research* |  |
|  | *161* | *T2 Research: Translation to patients* | *Phase 2 and 3 clinical trials controlled studies leading to clinical application evidence-based guidelines;* |  |
|  | *162* | *T1 Research: Translation to humans* | *Proof of concept studies  Phase 1 clinical trials New methods of diagnosis, treatment, and prevention in highly-controlled settings* |  |
|  | *163* | *T0 Research: Basic biomedical research* | *Preclinical and animal studies, not including interventions with human subjects* |  |
| 164 | | NIEHS Translational Framework: map the study to the most relevant part(s) of the framework. (select all that apply) |  | Work down from Impact to Fundamental Questions. When one fits the paper, select that and stop. Exposure studies are usually fundamental questions (unless if synthesis article or biomarker validation). |
|  | *165* | *Impact: assess the impact of a practice, guideline, or policy* | *Change in clinical outcomes Change in economic outcomes Change in environmental exposures Change in population outcomes* | Assess the impact of a practice, guideline, or policy: “so what? At a big picture level, what changed as a result of the research?” |
|  | *166* | *Practice: moving established and validated ideas into common practice to affect change among a broader population* | *Research practice Clinical practice Public health practice Individual behaviors Policy Risk management* | Moving established and validated ideas into common practice to affect change among a broader population: “taking what was learned and disseminating it more broadly, so that something is taken up by those in research, public health fields, the clinical environment, etc.” |
|  | *167* | *Implementation & Adjustment: testing in real world settings and adjusting for differences in settings and populations* | *Clinical testing Biomarker, screen, assay validation Effectiveness/Value Analysis Tool validation/optimal use testing  Intervention validation  Risk assessment* | Testing in real world settings and adjusting for differences in settings and populations: “does the intervention work in the messiness of the real world, in different clinical settings, for different groups of people, when we take it out of the highly controlled trial environment? What do we need to modify/adapt to different settings or populations?”  Risk assessment studies should say that they are doing a risk assessment or finding predictive risk (not just finding the association between a risk factor and disease). An example would be using a risk calculator and asking what the 10 year risk of getting a disease is. |
|  | *168* | *Application & Synthesis: testing in a structured and predictable setting to gain deeper understanding of a process or a particular effect* | *Intervention pilot testing Method testing Replication Tool testing Other controlled testing Research synthesis* | Testing in a structured and predictable setting to gain deeper understanding of a process or a particular effect: “when we control as many of the variables as possible, do we see a certain effect?” |
|  | *169* | *Fundamental Questions: Identification, observation, understanding* | *In Silico Organism In Situ Organism In Vitro Organism Ex Vivo Organism In Vivo Organism  Group and Population Organism* | Identification, observation, understanding: “before testing something in a structured setting, what foundational studies do we need to do to better understand the topic under investigation?”  i. In silico Organism: computer simulation/modeling ii. In Situ Organism: a specific protein or gene that is studied in its original “position”/location within an organism iii. In Vitro Organism: a cell or part of a cell, studied in a test tube, culture dish, or otherwise outside a living organism iv. Ex Vivo Organism: organ or tissue isolated from the rest of the body v. In Vivo Organism: a living organism vi. Group and Population |
| 170 | | Please point out any major issues encountered when applying the form to this study. |  | Please use very sparingly. |
